# Early Intervention with a Parent-Delivered Massage Protocol Directed at Tactile Abnormalities Decreases Severity of Autism and Improves Child-to-Parent Interactions: A Replication Study

**DOI:** 10.1155/2015/904585

**Published:** 2015-03-24

**Authors:** Louisa M. T. Silva, Mark Schalock, Kristen R. Gabrielsen, Sarojini S. Budden, Martha Buenrostro, Gretchen Horton

**Affiliations:** ^1^Teaching Research Institute, Western Oregon University, 345 N. Monmouth Avenue, Monmouth, OR 97361, USA; ^2^Providence Neurodevelopmental Center for Children, St. Vincent's Hospital, Portland, OR, USA; ^3^Sikhara Group, 3434 NW Savier Street, Portland, OR 97210, USA

## Abstract

Tactile abnormalities are severe and universal in preschool children with autism. They respond well to treatment with a daily massage protocol directed at tactile abnormalities (QST massage for autism). Treatment is based on a model for autism proposing that tactile impairment poses a barrier to development. Two previous randomized controlled trials evaluating five months of massage treatment reported improvement of behavior, social/communication skills, and tactile and other sensory symptoms. This is the first report from a two-year replication study evaluating the protocol in 103 preschool children with autism. Parents gave daily treatment; trained staff gave weekly treatment and parent support. Five-month outcomes replicated earlier studies and showed normalization of receptive language (18%, *P* = .03), autistic behavior (32%, *P* = .006), total sensory abnormalities (38%, *P* = .0000005), tactile abnormalities (49%, *P* = .0002), and decreased autism severity (medium to large effect size, *P* = .008). In addition, parents reported improved child-to-parent interactions, bonding, and decreased parenting stress (44%, *P* = .00008). Early childhood special education programs are tasked with addressing sensory abnormalities and engaging parents in effective home programs. Until now, they have lacked research-based methods to do so. This program fulfills the need. It is recommended to parents and ECSE programs (ages 3–5) at autism diagnosis.

## 1. Introduction

Autism spectrum disorder (ASD) is the most rapidly growing childhood developmental disability in the United States [1]. Currently, it is estimated to affect one in 68 children [2]. To date, there is no known cause, no known cure, and no known explanation for why symptoms emerge prior to the age of two. In 2013, the diagnostic criteria for ASD were updated and abnormal sensory responses were included [3]. Today, ASD is diagnosed by a dyad of persistent symptoms that arise before the age of two: deficits of social interaction and restricted, repetitive behaviors, including sensory abnormalities. Symptoms occur on a spectrum of severity that is characterized on the basis of communication deficits and inflexibility of behavior.

Abnormal sensory responses were included in the diagnostic criteria for autism in 2013 because there was mounting evidence that they are directly related to severity of autism [4]. They occur early in life, encompass a range of hypo- and hyperreactivity, and are highly prevalent in children with autism [5]. Since early in the research literature, abnormal sensory responses have been widely thought to be due to disordered processing of sensory information [6–10]. Of all the sensory abnormalities, abnormal tactile responses are the most prevalent, with reported rates over 95% by parent report and direct observation [11, 12]. They are described in terms that are remarkably severe and unusual [13] but have never been subjected to full neurological evaluation.

The neurological evaluation of tactile symptoms proceeds by evaluating sensory nerve function relative to touch, pain, temperature, vibration, and position in symptomatic areas and then ruling out central pathology [14]. Ruling out peripheral sensory impairment is done first because sensory loss early in development results in both disordered sensory processing and changes in brain structure [15, 16]. Surprisingly enough, this evaluative sequence has not been followed in autism, and to date only three small studies evaluating partial aspects of touch have been published [17–19]. As such, a primary impairment of touch has not been ruled out, and a full neurological evaluation of the sense of touch has been urgently called for [20]. The missing information is extraordinarily germane because touch is the primary sense informing the bonding and preverbal communication that is required for social and language development [21, 22], and impairment of the sense of touch early in development can readily account for the social/language delay and disordered sensory processing that has been so well documented in autism.

Over the course of 14 years and 10 peer-reviewed research studies, our research group has investigated a model and treatment for autism proposing that autism symptoms are due in some part to tactile impairment [12, 23–27] and are treatable with a massage protocol based on Chinese medicine. Loss/damage to the sense of touch is proposed to block the child's perception of soothing and affectionate parent touch and interfere with social/language and self-regulatory development. This can first be observed when soothing touch fails to soothe the child, and affectionate touch fails to stimulate the social response, simultaneous orienting, eye contact, and listening [28]. The tactile barrier to soothing is proposed to result in prolonged episodes of elevated sympathetic tone [29, 30] and account for the remaining sensory abnormalities on the basis of poorly regulated sympathetic tone disorganizing sensory thresholds [31, 32].

For the purposes of our research we developed a checklist of common sensory symptoms in children with autism that could be used to identify skin areas in need of massage treatment and to measure tactile and other sensory outcomes [33]. The checklist also identifies delays of early touch-stimulated self-regulation milestones, including self-soothing, attention, sleep, digestion, and behavioral self-regulation [34]. The validation study distinguished children with autism from typical and otherwise developmentally delayed groups by a multifocal tactile abnormality characterized by signs of painful withdrawal from noninjurious touch (allodynia/pain) and lack of response to injury (hypoesthesia/numbness) [33]. Ninety-three percent of the ASD group had signs of pain with touch on the face and in the mouth, 93% on the scalp, and 88% on the fingers and toes. Sixty-five percent had signs of numbness in response to injury. In addition, there was global delay of first-year self-regulation milestones. Pain and numbness are typical of the clinical presentation of small fiber neuropathy, a common disorder of small sensory fibers in the skin [35].

Experimental data from two randomized controlled trials (RCT) evaluating five months of daily massage treatment support our model for autism [24, 25]. The protocol is called Qigong Sensory Treatment (QST) massage for autism. The protocol is designed to increase circulation to the skin, normalize the child's perception of touch, and allow touch-stimulated social and self-regulatory development to unfold naturally in the context of the care and feeding of the child. The outcomes of the two studies, one evaluating a program of five months of dual parent- and therapist-delivered treatment (*n* = 46, QST Dual program) and the other evaluating the parent component (*n* = 47, QST Home program), showed increased social and self-regulatory abilities, reduced autistic behavior, and reduced tactile and other sensory symptoms. The intervention was effective in both high- and low-functioning children and ten-month follow-up indicated that continued treatment resulted in continued improvement [25]. Longer-term studies to determine the endpoint of treatment have not yet been conducted.

From the start of the research, it was evident that treatment outcomes were highly dependent on parent fidelity with daily treatment. However, children's initial resistance to touch, the range of tactile responses seen, the complexity of adjusting treatment to changing responses, and the need for long-term daily treatment called for a manual with flexible procedures and a program of ongoing parent training and support. Thus a five-month parent training program was developed, comprised of a parent handbook [36] and an initial 3-hour training, followed by 20 one-on-one support visits with a trained professional. This proved to be adequate to ensure fidelity with the intervention in the first five months of treatment.

The present study is part of a three-year, multisite RCT replicating and extending previous studies and evaluating the efficacy of the QST Dual massage intervention on tactile abnormalities and severity of autism in a larger group. In the first two years, treatment outcomes for 103 children under the age of six will be evaluated. In year three, treatment outcomes for 35 children between the ages of 6–11 will be evaluated. We will also seek further evaluation of tactile abnormalities with skin biopsy.

The three-year study will test two hypotheses.

1. Treatment of 103 preschool children with autism with the QST Dual massage intervention will result in decreased severity of autism across language, behavioral, and other sensory aspects of autism compared to the control condition. Treatment will be effective in both lower- and higher-functioning children. In addition, treatment will improve tactile and other sensory abnormalities and early self-regulation milestones and reduce parenting stress.
2. Treatment outcomes will be independent of severity of autism.

The objectives of this portion of the study are to evaluate initial 5-month treatment outcomes on severity of autism and on parent-child interaction and bonding. As compared to previous studies, this study will add new measures evaluating the overall severity of autism and receptive/expressive language level, as well as qualitative analysis of parent comments on parent-child bonding and interactions. The main outcomes are on measures of severity of autism, autistic behavior, and language development. The secondary outcomes are tactile and other sensory abnormalities, self-regulatory delay, parenting stress, and general development. In addition, qualitative data on parent comments will be presented and analyzed.

## 2. Materials and Methods

### 2.1. Study Design

This study was a multisite, randomized, single-blind, controlled trial of the QST Dual massage intervention in 103 preschool children with autism. Blinded professional examiners evaluated baseline and interval measurements. Baseline and interval reports were collected from parents. This is a replication and extension study of previously published studies; therefore, it was not possible for parents to remain blind to group. However, parents are known to be accurate reporters of current, observable child behavior [37] and parent information was treated as reliable and accurate. Children were randomized to treatment and control groups using a random number generator. Both groups were enrolled in early intervention programs. The treatment group received five months of daily parent-delivered massage and 20 sessions of therapist-delivered massage. Fidelity was assured with a formalized training program and ongoing support. The control group was in the waitlist condition and received treatment after five months. Treatment was directed towards normalizing responses to touch. Pre- and posttesting were done by the same professional examiners who were blind to group. Additional data was collected from parents. The study was conducted with Institutional Review Board approval and registered with the U.S. National Institutes of Health clinical trials registry (# NCT01801696).

### 2.2. Participants and Recruitment

Recruitment was accomplished via distribution of brochures, emails, listserv messages, website postings, presentations, social media, TV, radio, and word of mouth. In addition, invitation letters were sent to parents of children aged 2 to 5 receiving autism services from state-funded, early childhood special education and early intervention programs in eight counties in Oregon. Criteria for entry into the study included age of 2 to 5 years; diagnosis of autism; no additional chronic disability; no psychoactive medication or pharmaceutical chelation therapy; and not receiving more than fifteen hours per week of intensive behavioral treatment for autism. This criterion was chosen because there is no research evidence that low-intensity ABA treatment produces therapeutic outcomes [38, 39]. Only two study participants were identified as receiving ABA treatment; they were receiving 8 and 10 hours/week, respectively. Parents agreed to give their children the daily massage treatment for the duration of the study; to follow through with all training and support visits; and not to begin additional interventions for autism during the study. Parents provided records documenting their child's autism diagnosis. The majority of diagnoses had been previously made at autism evaluation centers using instruments such as the ADI and/or the ADOS. Children subsequently underwent a second diagnosis with such instruments prior to acceptance into early intervention autism programs. Prior to acceptance into the study, records were reviewed and the previous diagnosis of autism was confirmed. In addition, a developmental pediatrician reviewed the pretreatment testing and reconfirmed the autism diagnosis using DSM-IV criteria.

### 2.3. Study Completion

**Figure 1:**
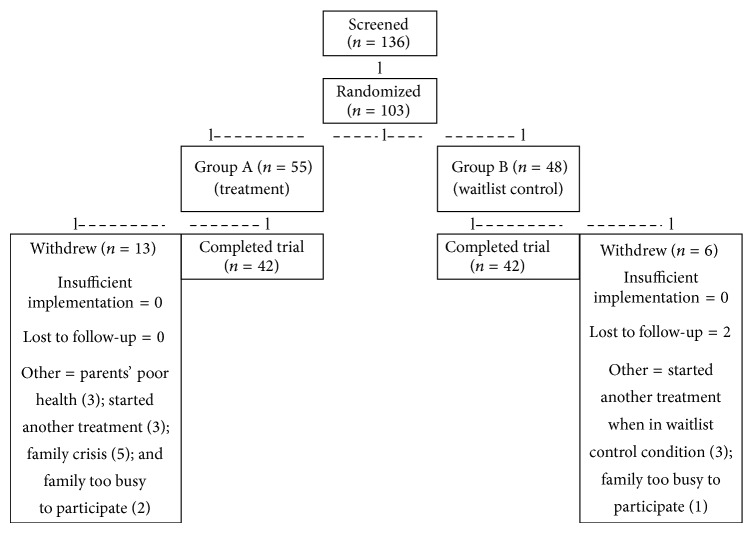
Participant flow diagram.

Figure 1 provides an overview of participant flow. One hundred thirty-six children were screened for the study during the period of September 2012 through April 2014 by the principal investigator and the project director. An additional 39 parents indicated interest in enrolling their children but did not submit enrollment documents. One hundred three children from 10 different counties in Oregon were determined to be eligible for the study, of which 55 were randomized to the treatment group and 48 to the waitlist control group. During the study period, 19 participants withdrew from the study for a total participant completion rate of 82% and a final *n* = 42 for both study and control groups.

### 2.4. Randomization Procedures

Children and their families from each geographical area who met study criteria were randomly assigned into either the treatment or control group condition based on age in months to reduce bias on developmental measures. A random number generator was used. One pair of siblings was assigned into the same group by necessity. Odd numbers of participants in each site resulted in uneven initial group sizes when randomized.

### 2.5. The Massage Protocol

The QST massage protocol is a whole-body massage that takes about 15 minutes to give and is usually done at bedtime. It is formalized in a parent training handbook with flexible constraints [36]. The parent does not avoid areas that are uncomfortable but instead works with them by attuning the massage techniques to the child's responses, within the comfort zone of the child. Over the course of treatment, tactile responses undergo predictable changes from hyposensitive to hypersensitive to normosensitive [36]. The protocol requires adjustment of the manual technique with each transition. The protocol also aims to sequentially stimulate social and self-regulatory activity, first by stimulating awareness and receptivity to massage, then by stimulating eye contact and smile, and finally by stimulating deep relaxation with touch.

The protocol has 12 parts that follow the acupuncture channels down the front and back of the body. Massage is carried out in a downward direction towards the hands and feet in the direction of capillary blood flow. Both patting and pressure are used according to the child's response. Generally, a quicker, lighter, patting technique is used to begin with, but in areas where the child withdraws from touch or is ticklish, slower pressing techniques are used. Additional options are available when neither patting nor pressure resolves the difficulty. For a summary of the massage movements, go to http://qsti.org/wp-content/uploads/2014/06/12MovementsAutism.pdf.

Therapists providing the parent training and support program benefitted from a 60-hour training. A total of 19 therapists participated in the study. Parent training unfolded with a group training followed by weekly one-on-one support. At each visit, therapists inquired about fidelity with daily massage, provided ongoing support and training, and gave children a massage treatment.

There are four time periods when parents are at risk for discontinuing the program. These are (1) upon initiating the program and not knowing how to deal with the child's resistance to massage; (2) during the transition period when the sensory system switches from hyposensitive to hypersensitive and massage techniques must be modified; (3) the transition period when the child moves into the autonomy phase of development and parenting techniques must be modified; and (4) the period after which the child has come to relax and enjoy the massage, progress is no longer dramatic, and daily massage can fall off the priority list. Therapists were instructed to watch for these at-risk periods and provide the necessary support.

### 2.6. Measures

Demographic information was obtained from participants and is shown in Table 1. Treatment and control groups were comparable. Overall, the percentage of boys (89%) was higher than the national average. Lower income families were identified as being at or below 100% and 150% of federal poverty guidelines for 2014 [40]. Representation of lower income families (53%) and culturally diverse families (44%) was higher than state demographics (Hispanic 34%, American Indian/Alaska Native 7%, and Native Hawaiian/Pacific Islander 3%). Eighty percent of parents involved had no previous experience with massage or Chinese medicine. Families who withdrew from the study did not have different demographics.

The following measures were used to evaluate five-month treatment outcomes.

*(1) Childhood Autism Rating Scale, 2nd Edition, Standard Version (CARS2-ST) [41]*. The CARS2 standard version is validated for children younger than 6. It has been found to be stable in the face of change over 12 months and not generally used as an outcomes measure. Total CARS scores range from 15 to 60. A score of 30 serves as the cutoff for a diagnosis of autism on the mild end of the autism spectrum; 30 to 36 is scored as mild to moderate; 36 and higher is scored as severe. The pretreatment median score of 39 was used as the criteria in the analyses to determine effects on language development by level of severity. Internal consistency is reported at .93, interrater correlation at .95, and test-retest stability at .88. The CARS2-ST was administered by professionals who were blind to group.

*(2) Preschool Language Scale, 5th Edition (PLS-5) [42]*. The PLS-5 has standardized subscales which evaluate relative ability in receptive and expressive language in children under 7. The test-retest stability coefficients ranged between .97 and .98 for the subscale scores and .97 to .98 for the total score. Internal consistency (split-half reliability) ranges from .91 to .93 for the subscale scores and .95 for the total score. The PLS-5 was administered by blinded professionals.

*(3) Vineland Adaptive Behavior Scales, 2nd Edition (Vineland-II) [43]*. The Vineland-II is a validated parent interview that assesses socialization, communication, motor skills, and daily living skills. Internal consistency split-half reliability coefficients were .97 for the composite scale and ranged from .83 to .95 for the domains. Test-retest coefficients were .94 for the composite scale and ranged from .88 to .92 for the domains. Raw scores were used in the statistical analysis while age equivalent scores are used to illustrate the magnitude of the changes in development over the treatment period. The social and daily living subscales were administered by blinded professionals.

*(4) Autism Behavior Checklist (ABC) [44]*. The ABC is a validated measure of autism and a component of the Autism Screening Instrument for Educational Planning. The ABC is used as a measure of change in response to the classroom program and as an outcomes measure in research. It measures behaviors typical of autism in five domains: sensory, relating, body and object use, language, and social and self-help. Adequate reliability and an alpha coefficient of .89 are established. Parents completed the ABC to report outcomes in the home setting at baseline and 5 months. The mean value for typically developing children is reported at 17.81 [45].

(Note: we were particularly interested to see whether we could replicate previous results in individuals with low functioning autism as there is little available evidence-based treatment for that group. Thus, we used two outcomes measures of autism severity: the CARS, a highly stable measure that is stable over a 12-month interval, and the ABC, a more sensitive questionnaire that is currently used in conjunction with a direct observation measure for evaluating educational outcomes [44]. Both the CARS and the ABC measure current functioning relative to similar aspects of autism but differ as to the length of time over which the measurement is taken. One is a direct-observer measure taken at a single point in time; the second is a questionnaire that solicits information describing the child at the present time. Thus, because child behavior can differ from day to day, measures were analyzed together in order to get a more balanced view of present functioning.)

*(5) Sense and Self-Regulation Checklist (SSC) [33]*. The SSC is a validated parent/caregiver measure of sensory difficulties and self-regulatory delays in children with autism under the age of 6. It is used as an outcomes measure in clinical practice and research. Scores can be generated for sensory and self-regulation domains. An overall internal consistency alpha of .87 has been demonstrated. Sensory items have an internal consistency alpha of .83; self-regulation items have an internal consistency alpha of .78. The SSC significantly differentiates children with autism from otherwise developmentally delayed (other DD) and typically developing (typical) children. The means and standard deviations for abnormal sensory response for ASD, other DD, and typically developing children are 39.6 (SD 10.6), 30.6 (SD 7.9), and 18.4 (SD 9.5), respectively. The mean oral/tactile scores for ASD, other DD, and typical children are 29.2 (SD 7.9), 22.8 (SD 6.7), and 13.5 (SD 7.2), respectively. The mean self-regulatory difficulty scores for ASD, other DD, and typical children are 56.8 (SD 14.1), 44.0 (SD 13.4), and 25.8 (SD 11.3), respectively. The SSC was completed by parents and used in data exploration of intervening variables affecting treatment outcomes.

*(6) Autism Parenting Stress Index (APSI) [46]*. The APSI is a validated parent/caregiver measure of parenting stress relative to autism symptoms. It is intended to measure the level of difficulty experienced by parents in successfully parenting the various physical and behavioral manifestations of autism, as well as factors impacting parenting stress such as lack of feeling close to the child, and concerns about the future of the child. It is used as an outcomes measure in clinical practice and research. Current data indicate adequate internal consistency (.83) and test-retest stability (.89). A validation study comparing scores from children with ASD with normally developing and otherwise developmentally delayed peers showed that the scales differentiate significantly between groups, with mean scores for ASD, other DD, and typical children of 23 (SD 10.4), 11.74 (SD 6.7), and 5.4 (SD 5.1), respectively. The APSI was completed by parents and used to measure changes in parenting stress relative to autism symptoms.

*(7) Beach Center Family-Professional Partnership Scale [47]*. The partnership scale is a validated tool that assesses satisfaction with services received. It contains two subscales: child-focused relationships and family-focused relationships. This 27-item scale is designed to be used as a research tool. Cronbach's alpha is 0.93. Parents completed the partnership scale at posttesting.

*(8) Fidelity and Social Validation Testing*. Therapists monitored parent fidelity with massage procedures by testing parents at weeks one and two with a checklist. The principal investigator monitored therapist fidelity with the parent training and support program. Parents completed a daily log recording fidelity with daily massage, reasons for missing the massage, and problems or concerns. In addition, parents completed a posttreatment survey consisting of a series of open-ended questions exploring their reactions to treatment and outcomes. See Table 2 for posttreatment survey questions.

### 2.7. Data Collection

Pre- and postintervention data collection was conducted within a one-month window both prior to beginning of treatment and after the 20-week intervention for children in both the treatment and control conditions. Parents completed an online set of surveys and background questionnaires that included the Autism Parenting Stress Index, the Sense and Self-Regulation Checklist, and the Autism Behavior Checklist. The Vineland-II, CARS2-ST, and PLS-5 were administered in the home by trained, blind-to-condition professionals. Treatment fidelity was monitored throughout the 20-week intervention by assigned therapists.

### 2.8. Data Analysis

Initial analyses were conducted to detect any potential attrition bias using 2-way ANOVA and MANOVA on preassessment outcome measures. This was followed with analyses to confirm equivalence of treatment and control groups on preassessment outcome measures using 2-way ANOVA and MANOVA. Descriptive and paired *t*-tests on outcome measures were conducted for both treatment and control groups. Main treatment effects were tested using 2-way repeated measure ANOVAs and MANOVAs. Testing whether treatment outcomes were independent of language ability and severity of autism was also conducted using 2-way repeated measure ANOVAs and MANOVAs.

## 3. Results

### 3.1. Sample Size Justification and Power Analysis

A power analysis to determine sample size for this study was conducted using pooled data from all children under the age of six who had participated in our previous studies and received the QST intervention. The primary outcome used was the composite score from the Pervasive Developmental Disorders Behavior Inventory [48]. An *n* of 45 for both the treatment and control group, with 12% attrition, yielded a final *n* of 40 for each group. Allowing for attrition, a final *n* of 40 in each group, and a *P* < .05, a power analysis yielded a power of .99 on parent measures.

### 3.2. Potential Attrition Bias

Participants in the control and treatment condition withdrew at a proportional rate *X* ^2^  (1, *N* = 100) = .841, *P* = .359. Two-way ANOVA and MANOVA indicate no differences between completers and noncompleters on outcome measures. *F* values range from .018 to 3.57 with associated *P* values ranging from .894 to .062. There was a difference in age between completers and noncompleters, with noncompleters being on average six months younger than completers *F*(1,98) = 4.58, *P* = .035.

### 3.3. Equivalence of Treatment and Control Groups

Participants in the treatment and control conditions did not differ on outcome measures or age. Two-way ANOVA and MANOVA indicate no differences between groups on outcome measures and age. *F* values ranged from .619 to 1.56 with associated *P* values ranging from .528 to .216.

### 3.4. Preassessment to Postassessment Changes

Table 3 displays descriptive pre- and postoutcomes for both treatment and control groups. Paired *t*-test results are also shown. Treatment group participants experienced significant improvement on all measures. Control group participants also experienced significant improvements on a number of measures, although typically these changes were not as large as for the treatment group. Because mean scores for text typically developing children are well above zero, an additional column calculating percent (%) change toward typical norm has been added to Table 3 to show the percent (%) normalization of the reported change. Children in the treatment group experienced a 38% decrease in abnormal sensory response toward normalization. These children experienced a 49% decrease in abnormal oral/tactile response toward normalization. The decrease in self-regulatory difficulties represented 34% improvement toward normalization. Autistic behavior decreased 32% towards normalization. Parents of these children experienced a 44% decrease in stress toward normalization.

### 3.5. Intervention Effects on Main and Secondary Outcomes

Main outcomes include severity of autism, language, and general development. Secondary outcomes include sensory, self-regulation, and parenting stress.  Table 4 presents results from the 2-way repeated measures ANOVA and MANOVA analyses. Equality of error variances were confirmed for all analyses. There was an overall treatment effect in reducing the overall severity of autism in the treatment group as measured by the CARS and ABC. Effect size was in the medium to large range. Post-hoc univariate analyses found a significant effect on the ABC but not the CARS. The correlation between the ABC and CARS was low (.273), indicating that they measure different things.

Separate 2-way repeated measure ANOVAs were conducted on receptive and expressive language. A significant treatment effect was found on receptive language with an effect size in the medium range. No treatment effect was found on expressive language.

A 2-way repeated measures MANOVA was conducted on general development of social and living skills. No treatment effect was found on general development. Both groups experienced increases in raw scores equivalent to 4–8-month increases in mental age on living skills and 4–6 months on socialization.

A 2-way repeated measures MANOVA was conducted on sensory and self-regulation. An overall significant treatment effect was found with a large effect size. Post-hoc univariate analyses found a significant effect on the sensory abnormalities, tactile/oral abnormalities, and the self-regulatory difficulties.

Finally, a 2-way repeated measure ANOVA was conducted on parenting stress. A significant treatment effect was found with a large effect size.

### 3.6. Effectiveness of Treatment by Severity of Autism

To further test whether the effectiveness of the treatment was independent of severity of autism, the treatment group was split into mild/moderate (a score of less than 39 on the CARS) and severe (a score of 39 and higher on the CARS) autism subgroups. Paired *t*-tests were performed on the outcome measures for the subgroups.

As can be seen in Table 4, there was an overall significant treatment effect on receptive language. Further, children with both mild/moderate and severe autism experienced significant improvements in receptive language as measured by the PLS-5 Auditory Language (*t* values are −4.26 and −4.29, respectively, with associated *P* values of .0001 and .0003).

The results in Table 4 show overall significant multivariate and post-hoc univariate treatment effects on abnormal sensory response and self-regulation. Again, children with mild/moderate and severe autism both experienced significant reductions in abnormal sensory response and self-regulatory difficulties as well as tactile/oral abnormalities (*t* values ranged from −4.24 to –5.88, with respective *P* values ranging from .0004 to .00001).

The overall significant treatment effect on reducing parenting stress is shown in Table 4. Parents of children with mild/moderate and severe autism experienced significant reductions in stress (*t* values were −5.135 and −4.448, with associated *P* values of .0001 and .0002).

### 3.7. Parent Satisfaction and Reactions to the Treatment and Outcomes

The Beach Center Family-Partnership Scale survey showed that 95% of the treatment group parents were very satisfied with their experience with the intervention, and 5% were somewhat satisfied.

Table 5 lists examples of parent responses to posttreatment survey questions about the parent experience of changes in their child. The table has additional columns reporting the survey question referenced (see Table 2). The table illustrates the normalization of touch and relationship that all parents experienced in one way or another with their child and gives examples of the accompanying sensory and behavioral changes that they observed.

Table 6 lists the types of responses parents had to posttreatment questions 4 and 5: “When you compare the massage to other treatments, how does it differ?” and “If you could sum up your experience in a way that would be helpful for another parent considering this treatment, what would it be?” Themes that emerged were improved bonding (18), parent empowerment to actively help their child (12), treatment supporting overall development (5), and ability to easily give the treatment at home (3).

### 3.8. Fidelity

Therapists tested parents on the 12 massage movements after the first and second week of treatment using a checklist. At the end of the second week, 75% of parents demonstrated complete fidelity with massage procedures. The other 25% needed correction on one or two of the 12 parts of the massage. For those that did not complete all 12 movements correctly, therapists checked on fidelity each of the following weeks until all 12 movements were correct. Twenty-nine of the 42 sets of parents provided the treatment daily throughout the 5-month treatment period. The balance, 13, provided the treatment less than daily during some periods, usually due to sickness of the child or the parent. All therapists provided 20 training and support visits.

### 3.9. Adverse Effects

There were no adverse effects reported in children. One parent with severe wartime PTSD found that he was unable to give the massage due to excessive anxiety triggered by his child's resistance to touch. Once he stopped giving the massage, he experienced no further anxiety relative to the massage.

## 4. Discussion

This is the first evidence-based intervention for tactile abnormalities in children with autism that effectively treats them with a massage protocol rather than making environmental accommodations to them. Before treatment, the mean tactile abnormality score for the treatment group was more than twice that of the typically developing group. After treatment, scores normalized by 49%. Parents reported that the massage helped to build a stronger bond and improved the experience of touch and relationship. Children sought out touch and affection from their parents, and parents felt closer and more connected. Child-to-parent attachment difficulties are described in the autism literature [49], and the profound degree to which they impact the parenting experience is illustrated in the parent comments, the very large decrease (44%) in parenting stress, and the very high parent satisfaction rate (95%) when they improve. Attachment theory has had difficulty in accounting for attachment difficulties in autism because they exist despite evidence of normal parent-to-child bond and sensitivity [50]. We suggest that normal attachment requires child sensitivity as well and that tactile abnormalities interfere with child-to-parent bonding. The very good news is that tactile abnormalities are reversible with treatment.

We were particularly interested in the 5-month treatment outcomes on receptive language. Concordant with increased diagnosis of high functioning autism (HFA) [51], this study had a higher proportion of children with HFA than previous studies. In children with low functioning autism (LFA), receptive language delay stands in the way of the development of speech; in children with HFA, receptive language delay manifests as echolalia or as monologues on topics of interest, for example, dinosaurs or robots. As it turned out, receptive language improved in both groups by a mean value of 18%. We ascribe the improvement to the effect of normalization of touch on foundational social and nonverbal communication abilities: the ability to be receptive to another person, make eye contact, give face-to-face attention, and listen. All of these are normally stimulated by touch in the first year of life in typically developing children but tend to be absent in young children with autism.

This was a replication and extension study, a necessary step before an intervention can be more widely recommended. Results replicated earlier studies with regard to overall decrease in severity of autism, improvements in autistic behavior, communication, sensory symptoms, and parenting stress. As shown previously, treatment was effective in both low- and high-functioning children. We were particularly interested to see whether we could replicate previous results in individuals with LFA, as there is little available evidence-based treatment for that group. We used two outcomes measures of autism severity: a highly stable measure that is stable over a 12-month interval (the CARS) and a more sensitive questionnaire currently used in conjunction with a direct observation measure for evaluating educational outcomes (the ABC). Thus it was quite remarkable that the CARS confirmed the findings of the ABC in showing significant decrease in severity of autism in the LFA group over the relatively short treatment period of five months. The CARS did not reflect the improvement seen on the ABC in the HFA group. We think this is most likely because the CARS was not standardized on individuals with HFA, has a compressed range of scores for HFA, and is less sensitive in that group [52]. We await the one- and two-year results to see whether the CARS will confirm the ABC results in the HFA group after a longer intervention period.

Taken altogether, these results represent a breakthrough in autism treatment. It is the first research-based intervention to be effective for the individual sensory, behavioral, and language components of autism as well as severity overall; it is the first intervention to be effective in both lower- and higher-functioning children. And it is the first time a model for autism revolving around tactile abnormalities is presented and supported with experimental data. The intervention was powerfully effective with children, because parent touch is critical to early social and self-regulatory development and because the intervention normalized children's sense of touch. As a result, behavior improved, social and self-regulatory gaps in the developmental foundation are filled in, and development was better supported.

Early intervention policy is to involve parents of children with autism in the direct care of their child's disability and offer them training and support in research-based home interventions. Parents desperately need effective tools to help their children. Two parent-delivered interventions are commonly recommended to parents of newly diagnosed children with autism by occupational therapists: the Wilbarger brushing protocol and joint compression [53, 54]. There is little research demonstrating efficacy on autism symptoms for either method. Since there is now replicated research to show efficacy for QST massage for autism, we recommend that parents be offered training and support in QST massage at the time of their children's autism diagnosis.

Over the past 14 years, our research has been conducted in collaboration with state-sponsored, early intervention programs for autism. School administrators are well aware of the importance of early identification and treatment of hearing and vision impairment to educational outcomes for children. A randomized controlled trial conducted in children with autism in early intervention classrooms has already shown that classroom behavior and social communication skills improved in the classroom with QST massage [25]. As the tactile abnormality in autism comes to be better understood and QST massage comes to be better known, we anticipate that tactile abnormalities will also be recognized as important to educational outcomes and that QST massage will be recommended. In our view, all children with autism should be evaluated for the need for hearing aids, glasses, and QST massage at the time of diagnosis. The results presented here support earlier recommendations to implement the QST program at the time of autism diagnosis concurrent with the early intervention program in order to prepare the child for school. If tactile and other sensory symptoms can be remediated prior to entering school, educational outcomes can only be enhanced.

## 5. Conclusions

The QST Dual program for autism directed at tactile abnormalities was effective in decreasing severity of individual sensory, behavioral, and language components of autism as well as severity of autism overall. The intervention works by decreasing tactile and other sensory abnormalities and removing the sensory barriers to learning social/language skills and regulating behavior. Child-to-parent bonding improved, and the experience of touch and relationship normalized for parent and child. Children were better able to make eye contact, focus, and listen, and parenting stress decreased. This program can be recommended to parents and early intervention programs at the time of autism diagnosis. It can be expected to improve educational outcomes for children and reduce stress in the preschool classroom environment.

## Figures and Tables

**Table 1:** Participant demographics.

	Tx active	Tx dropped	Tx total	Control active	Control dropped	Control total	Total active	Total dropped
Total number	42	13	55	42	6	48	103	19
Gender								
Male	36	12	48	39	5	44	92	17
Female	6	1	7	3	1	4	11	2
Race^*^								
AI/AN	4	1	5	1	1	2	7	2
Asian	4	0	4	5	0	5	9	0
NH/PI	0	1	1	2	0	2	3	1
Black	1	2	3	3	1	4	6	3
White	40	10	50	39	6	45	95	16
Other	4	2	6	1	0	1	7	2
Ethnicity								
Hispanic/Latino	11	4	15	7	2	9	24	6
Not Hisp./Lat.	31	9	40	35	4	39	79	13
Poverty level								
100%	9	1	10	9	1	10	20	2
150%	16	3	19	16	1	17	36	4
Did not share	4	3	7	6	0	6	13	3
income level								

Tx = treatment.

Tx drop = those participants who were in the treatment group condition that dropped out.

Control drop = those participants who were in the waitlist control group condition that dropped out.

AI/AN = American Indian/Alaska native.

NH/PI = native Hawaiian/Pacific Islander.

^*^Note: parents could select more than one ethnicity/race. So the numbers add up to more than the number of subjects.

**Table 2:** Parent posttreatment questions.

Question	
(1) What has the massage done for you and your child?	

(2) Compared to before starting this program, do you use touch more, less, or the same when your child is having behavior problems?	

(3) What changes have you seen in your child since beginning the massage?	

(4) When you compare the massage to other treatments, how does it differ?	

(5) If you could sum up your experience in a way that would be helpful for another parent considering this treatment, what would it be?	

**Table 3:** Preassessment to postassessment results for the QST massage.

	Treatment group (*n* = 42)						Control group (*n* = 42)					
	Preassessment mean (SD)	Postassessment mean (SD)	Diff.	% norm	*t*	*P*	Preassessment mean (SD)	Postassessment mean (SD)	Diff.	% norm	*t*	*P*
Parent measures												
Parenting stress	24.3 (9.51)	15.8 (8.9)	−8.5	44%	−6.49	.0000001	22.5 (9.33)	21.0 (9.6)	−1.5	9%	−1.39	.171
ABC	82.4 (25.9)	61.5 (26.6)	−20.9	32%	−5.64	.000001	83.1 (25.9)	75.7 (28.6)	−7.4	—	−2.48	.018
Abnormal sensory response	39.7 (9.1)	30.4 (9.8)	−9.3	38%	−6.68	.00000005	41.3 (10.3)	38.6 (11.6)	−2.7	11%	−3.00	.005
Tactile/oral abnormalities	29.6 (6.8)	22.4 (8.0)	−7.2	49%	−6.14	.0000003	29.6 (7.7)	27.7 (8.6)	−1.9	11%	−2.59	.013
Self-regulatory difficulties	57.6 (11.2)	45.1 (11.5)	−12.5	34%	−7.64	.000000001	57.4 (13.4)	54.0 (14.5)	−3.4	9%	−2.35	.024
Blinded assessor measures												
Vineland daily living skills	34.3 (17.7)	42.7 (19.1)	8.4		5.21	.00001	37.5 (20.0)	45.9 (22.7)	8.4		5.50	.000002
Vineland socialization	36.0 (14.4)	45.7 (16.3)	9.7		5.54	.000003	40.7 (17.4)	48.6 (21.0)	7.9		5.82	.000002
CARS	39.7 (6.6)	38.2 (6.6)	−1.5		−2.61	.013	38.0 (7.8)	37.7 (7.8)	−0.3		−1.13	.266
PLS-5 auditory communication	25.9 (12.1)	30.6 (12.1)	4.7		6.07	.0000004	29.2 (13.5)	31.8 (14.3)	2.6		5.16	.00001
PLS-5 expressive communication	26.5 (11.1)	30.0 (11.1)	3.5		6.22	.0000002	28.2 (11.6)	31.3 (13.1)	3.1		5.54	.000002

Note: SD = standard deviation; Diff. = the change from pre- to postscore; % norm = the percentage reduction in parenting stress, autistic behavior, abnormal sensory responses, and self-regulatory difficulties toward typically developing children.

**Table 4:** Treatment effects for QST massage (repeated measures Anova/Manova).

Variable	Group main intervention effect				
	*F*	(Degrees of freedom)	*P*	*η*_*P*_^2^	
Univariate analysis					
Stress	17.2	(1,82)	.00008	.173	Large
Receptive language (PLS-5 auditory)	4.81	(1,82)	.031	.055	Medium
Expressive language (PLS-5 expressive)	0.18	(1,82)	.667	.002	Small
Multivariate analyses					
Severity	5.17	(2,81)	.008	.113	Medium-large
Post-hoc					
ABC	8.11	(1,82)	.006	.090	
CARS	3.34	(1,82)	.071	.039	
Sensory and self-regulation	7.40	(3,80)	.00002	.217	Large
Post-hoc					
Sensory	15.16	(1,82)	.00002	.156	
Tactile/oral	14.92	(1,82)	.00002	.154	
Self-regulation	17.92	(1,82)	.00006	.179	
General development	0.42	(2,81)	.656	.010	Small
Post-hoc					
Vineland daily living skills Vineland socialization	No post-hoc analyses				

**Table 5:** Parent comments on changes in the child and changes in relationship.

ID #	Changes in the child	Q	Changes in relationship	Q
1	She is a lot more cuddly. Her sensory issues have improved a little	1	This has brought us closer together. She is more friendly with family members.	4

2	Increased calm, less tantrums, increased communication/speech. He is now potty trained!!!!	1		2

3	He doesn't have the long tantrums he used to have.	3	We've become closer and he's developed a stronger bond with both his parents. His speech development has made dramatic progress. He's using full sentences now, often to our amazement and gratitude.	1

4	He tolerates touching and the massage a great deal more. He now cries when he's hurt. He makes eye contact more often, and displays spontaneous affection way more than he used to!	3	We have developed a closer relationship, his development accelerated, and he is much more social than he was.	1

5	Meltdowns are fewer and less severe when they do occur, and he is using his words much more frequently.	3	He has become more interactive in class, even interacting with a few other students. He has taken the initiative to ask other kids to play with him at our local park.	3

6	We are able to cut his finger and toe nails with very little fuss! We've also seen some progress in potty training.	1, 3		

7	He wants us to touch him more. He's more talkative, learning to use the toilet more, gained a bunch of new skills.	3	He loves his massage. It calms him down, he likes spending the time with me. It makes him nice and sleepy before bed.	1

8	Touch seems comforting to my child after starting the program. Easy transition, less tantrum.	3	The most obvious one is the child get a lot closer to family members.	1

9	Some of his sensory issues are starting to shift for the better. More eye contact. He's more aware of what is going on around him. More tantrums from being more aware.	1	He is more cuddly and affectionate than before. Now he hugs and kisses his stuffed animals, as well as gives his dad and I more hugs and kisses.	3

10	He has started to self soothe. He tries new thing and is interested in more things than just matchbox cars. He has grown up so much. It is amazing to see the transformation. It's like the frustrated little boy who couldn't get his point across has finally come out and has so much to say and do.	1	I feel like it bonds the parent to the child. I think it opens the child up and they understand they are safe.	3

11	He makes better eye contact and will make eye contact when asked. He has developed some self-soothing techniques and has improved in his toileting.	3	He is socializing more with other children and will ask his sister to play. He uses his words to communicate a lot better than he had before the massage. He has better patience and self-control.	

12	She made a lot of eye contact, improving every week. Likes hugs and massages, calms her down.	1	Connection between father and daughter at night time.	1

13	Better interactions. Better for her as far as being comfortable being touched. Sleep has improved.	1	Better interactions. Massage builds a better bond.	4

14	I gave my child a haircut without a single scream. This is a huge change. He now will sit in a chair at home, wear a cape, verbally say “you cut my hair mommy” and then sit and hold still and giggle when the clippers tickle his neck.	3	He has made huge leaps and bounds in his social interaction with the world since starting the massage, He is beginning to notice other people and appropriately interact with them and reciprocate language. His ability to communicate his own needs has also increased an amazing amount. He is now requesting for items in full sentences the majority of the time as opposed to one or two word requests 60% of the time. I loved that the massage focused on him being comfortable in his own body.	1

15	Well right after we started we had an extremely difficult time for about 4 weeks with our son. My husband stuck with doing his massage and it seems to have paid off. Brenner asks for it! He also does the “massage” to us. That is huge in itself. Overall there is consistent improvement.	1	He likes massage with me (mom), super silly for dad. He communicates better. He does not react towards people as bad as he did before.	3

16			More joyful content pride self esteem.	3

17	He can feel pain now! It amazes me to see him cry for even the tiniest scratch or bump. He also has stopped biting me. He looks forward to the massage.	3	He actively hugs me if he thinks that I am sad.	3

18	He's more present. Massage helps me calm him.	3	Massage strengthens our connection.	1

19	He's more aware of his body, more eye contact. Massage gives tools to help calm him. We can use the massage to get him to sleep if we need to. Potty training progress.	3	Social games, increase in spontaneous language and longer sentences. Massage makes an emotional and spiritual connection between us.	3, 4

20	He is able to sleep during the night…his language has catapulted and he is less aggressive!	3	Massage is calming and brings you closer to your child.	4

21	He does more eye contact. He gets more concentred [sic]. He slept through all the night.	3	Massage get [sic] you and your kid closer. It's like time to relax and show love with each other.	4

22	I has [sic] been able to calm him down a little bit.	1	Had some great momma and son time.	1

23	It's probably improved his eye contact somewhat. He eats better and is not as sensitive to touching sticky things as he used to be. He is able to calm himself down much easier. He rarely has major tantrums. If he starts one, it's usually over within a couple of seconds. He also seems to be sleeping better and it's easier for me to clip his finger and toe nails than it used to be.	3	It's been a good time of connection. About half the time, the massage seems to help calm him down before bed.	1

24	It helped him transition before during and after the move.	1	It helped him get a bit more calmed after massage and he wanted to cuddle right after.	1

25	I can cut her nails while she's awake, She has tried some new foods that she used to eat when she was younger, her bowel movements are not as hard - there's practically no pushing on her end. She uses pronouns properly now, she uses a ton more words.	3	It's done by me and my husband, no outsider can do it better than we can. Every other improvement seems to be because of some professional, this is because of our touch.	4

26	His ability to calm himself or request aid in calming himself is unmatched, started taking an interest in potty training. Improved verbal and communication skills.	3	Brought us closer. We both really enjoy the time we spend doing the massage together. It's been a great bonding experience for us. It also is a good time for both of us to de-stress and calm down together.	4

27	Well I could not pinpoint one in particular.	3	Got me more in tune with my son's body language.	1

28	He moves his fingers and toes, explores them, and is responsive and ok with the feeling or sensations in them. He is toe walking less. He reacts to pain when he gets hurt. He sleeps most nights now. It used to be a couple hours. He is noticing when he is wet or poopy and will come to us to change him. He has more self awareness and self confidence. He is more engaged and will look at us more. Sometimes he will respond to his name being called. Often he will initiate a game with eye contact. He gets frustrated less often because he is able to physically do more things successfully. Seems to be interested in more foods.	3	Overall, he is so much happier, calmer, and life is so much more enjoyable and livable. He talks more, babbles all the time, and we can understand some words, once in a while will use one with purpose or meaning.	3

29	He is much calmer and affectionate. Overall he seems happier and is communicating much better. He is 100% potty trained and is much more social with children he sees frequently.	3	He has always cuddled with his dad, but has been very selective cuddling with me. Now he approaches me, wants me, asks for me, and it feels like he loves me whereas before it did not.	1, 2

30	Lots of big hugs, back patting, and hand holding - it helps with attention.	2	I feel like he has become more oppositional, but I think it's more that we're getting to know his limits and body language. At the same time, he's more cuddly than ever and wants to be held and hugged when he has an owie. At times he wants us to pat him to get the “jellybeans” out.	3

31	He is more present daily and seems to be opening up. He is sleeping much better. He is less over stimulated, he can handle going into crowed places and does not run away.	1	He is more receptive. We have bonded more. He will now spontaneously give us hugs and show affection. His speech is coming along.	3

32	Most changed would be he understands more.	3	With the massage he is more manageable daily to live with.	1

33	It has built more trust when it comes to touch.	3	She seems to be trying to talk more - making more verbal noises. Interested in what we are doing, trying to involve herself.	3

34	Happier and less fighting with other children. Has lowered violence at school.	1	Seems friendlier and more willing to interact.	1

35	We saw change right away upon beginning the massage. His language increased as well as his awareness of what was happening in his environment. He began to understand much more of what we were communicating to him & also began communicating more effectively with us as his language skills & comprehension grew stronger. His frustrations & tantrums decreased due to this improved communication & understanding. He has more eye contact & is much more responsive to questions and/or instructions. He seems to be more motivated to work harder at communicating with us & others. His focus has improved greatly.	3	He has developed a relationship with his sister. He has become very empathetic towards others. He is extremely protective of his sister, making sure she isn't too far behind and has even become very worried when she has a dirty diaper in public. The relationship he has with his older brother has gotten stronger. He addresses people that he knows well by name. He does “hi” and “bye” greetings often now.	3

36	He is becoming more comfortable with physical affection and comfort. All around vast improvements in every area that I can think of. Improvements in eating, sleeping, interactions with everyone around him, understanding and expression, toileting, concentration, appropriate play.	3	I feel it has brought us closer together both physically and emotionally. Before doing the massage therapy his dad and I made a point of being affectionate with him. We hugged him, kissed him, snuggled him as much as we could. He generally responded by “allowing” us to “assault” him…or at least that's how it felt. He would generally stare off away from us and he rarely responded in kind. After several months of massage he is reciprocating and will even seek hugs and comfort. I feel that is the most significant change, for us, since starting massage therapy.	1

37	I truly feel it has helped him be more “present” and aware of his surroundings. His speech has come incredibly far and potty training is starting to take off right now.	3	It has given us a way to encourage different kinds of touch. Added in a positive way to our bed-time routine.	

38	More eye contact and more verbal (more spontaneous communication but not a lot). Follows directions better and seems to pay attention and listen longer when I'm talking.	3	It's a practical way to engage and help your child.	3

39	She's able to self feed and won't spit out new foods.	3	Bonding	5

40	He is happier.	3	Massage will help parents and kids somehow get closer together.	3

41	In the beginning he made huge gains in speech especially (he never before seemed to be present at speech therapy or seem like he understood what was going on) and also things like being touched. I often reach for his head and his chest now to calm him. I often will just hold him when he's acting out.	3	That it helps the parents to actually bond with our kids and to pay close attention to small gains.	4

42	He is beginning to gained the ability to tolerate touch.	3	Got us closer, and I was able to understand him more.	1

**Table 6:** Parent comments on the intervention.

ID #	Parent comment on the intervention
1	This has brought us closer together.

2	More “active” form of therapy. Continue to stick with the massage and you will see improvements in your child.

3	I feel that it's accelerated his progress more than anything else.

4	This is something I can do at home at any time, and it's easy to do. It's also tailor-able to his individual issues. It really does make a difference, and it's so worth the time you put into it!

5	So far Qigong is the only treatment he's had that showed a significant difference in behavior.

6	Relaxing

7	Less invasive, less medical, more natural feeling

8	I would strongly encourage other parents to try it.

9	This is the only treatment he's received to date.

10	I feel like it bonds the parent to the child. I think it's opens them up and they understand they are safe.

11	He has had outpatient speech and occupational therapy as a toddler as well as services while in school. I think the massage has complimented these services.

12	Relaxes her to the point it is easy transition for bedtime.

13	Hands on builds stronger bond

14	I loved that the massage focused on strengthening his skills and working on him being comfortable in his own body.

15	That it is about touch and energy and doing something for him, not asking him to “do” something for us, that is, speech therapy.

16	More fun

17	He is non-verbal so the massage does not help with specific speech sounds. He pats his legs to show me he is ready for massage at bedtime. Qigong works!

18	I feel more empowered

19	Emotional and spiritual sort of connection. parent driven and consistent (daily). Awareness to body.

20	The massage is parent delivered…all the other methods for his autism are done by others…it is intimate and therefore I feel like I am taking an active part!

21	Massage gets you and your kid closer. It's like time to relax and show love with each other.

22	I can almost do his massage anywhere. We love our massage time.

23	We haven't really tried other treatments but what I like about the massage is that there is really no way to harm him by giving it to him. It's gentle, safe and fosters our relationships with him.

24	Well it was great that someone knowledgeable was performing it. My son beneficiated more from that than other massages where someone just tells you what to do but no one oversees your implementation.

25	It's done by me and my husband, no outsider can do it better than we can. Every other improvement seems to be because of some professional, this is because of our touch.

26	This is the first actual hands on treatment we have been offered. It's not more vitamins or shots, it's something he is involved in and really enjoys. It's not a “job” like ABA or the stuff he does at school, it's far less like traditional treatment.

27	Personal help. It's been eye opening

28	It is easy and something that I can do at home. It has been what makes all the other therapies work. It has done more for him than Speech and OT. It was like they didn't work until we started the massage.

29	It is easier, more calm and more bonding since you are touching your child the whole time. It is also more consistent.

30	Qigong massage gets you and your child in touch with their body and their needs. It gives you a way to communicate with your child about their needs.

31	It is something I can do for him and see the changes daily

32	We like it because we don't use pills. At first it is hard to do because it is something new. Stick with it because all you see is results.

33	This is really the only other treatment we have tried.

34	Lets you connect to your child and open up their senses and awareness.

35	This treatment gives our child a chance to relax & spend one-on-one time with us comfortably at home, as opposed to spending time with a therapist in a different setting.

36	It helped us break down the barrier between us and him. There was always this disconnect and none of the therapies we tried got us closer to him. Now he seeks us out for comfort and just to be close. If we sleep in on a weekend he'll come in and lay with us in bed rather than playing in his room alone. He comes pattering into our room and climbs into bed with us and will lay there until we get up or, if he decides it's been long enough, he'll start slapping our cheeks and saying, “Mama wake up, daddy wake up.” It's the most irritating thing in the world and we love it.

37	I like that the massage is every day like a medicine and it's easy to put into a routine. It has been a positive experience from start to finish and has given us nothing but rewards.

38	It's relaxing and It's a practical way to engage and help your child.

39	It's not pricey

40	Much less intrusive, more physical.

41	It's family oriented, it's long term, it's inexpensive, it has no risks. It makes you feel like you have some control in an uncontrollable situation.

42	That it helps the parents to actually bond with our kids and to pay close attention to small gains
